# 

**Published:** 1885-12

**Authors:** 


### OUR ADVERTISERS.

Always mention THE ATLANTA MEDICAL AND SURGICAL JOURNAL in writing to advertisers.

A PRIVATE INFIRMARY.—It affords us pleasure to recommend the private infirmary, for the treatment of diseases of women, of Drs. Taliaferro & Noble, of this city. It is a first-class institution in every respect.

COLDEN'S LIQUID BEEF TONIC.—Mark the changes in this advertisement in this issue. This preparation has the endorsement of the best men in the profession everywhere, and we think deservedly so. Send for sample and try it.

CELERINA—Dr. Jacob S. Phillips, of New York City, says: I beg leave to recommend Celerina as an excellent restorative after alcoholic excess; an aphrodisiac of exceptional merit, and an antispasmodic in hysterical manifestations and other functional nervous disorders.

M. J. BREITENBACH, NEW YORK.—This gentleman has an attractive advertisement in this issue of THE JOURNAL. Among the excellent preparations that he advertises we mention especially his extract of malt, condensed milk products and meat solutions. Try these goods and be convinced of their true worth.

FUCUS MARINA.—B. Roemer, M. D., of St. Louis, reports six cases of pronounced malaria in which he used the Elixir of Fucus Marina, prepared by the Peacock Chemical Company, in connection with quinine, with satisfactory results. He has also found its alterative action beneficial in syphilis, supplanting the usual prescribed smilax stillingia, or sarsaparilla.

